# The GET pathway modulates azole susceptibility through sterol metabolic remodeling and protein trafficking in filamentous fungi

**DOI:** 10.1128/mbio.02021-26

**Published:** 2026-10-07

**Authors:** Shuting Ye, Ziyu Cui, Mi Zhou, Chengcheng Hu, Pengju Yu, Long Zhang, Shaojie Li, Xianyun Sun

**Affiliations:** 1 State Key Laboratory of Microbial Diversity and Innovative Utilization, Institute of Microbiology, Chinese Academy of Sciences 85387, Beijing, China; 2 College of Life Sciences, University of Chinese Academy of Sciences 74519, Beijing, China; 3 National Institute for Radiological Protection, China CDC 12415, Beijing, China; 4 Shandong Jinniu Group Co., Ltd., Jinan, China; University of Wisconsin-Madison, Madison, Wisconsin, USA

**Keywords:** GET pathway, azole resistance, TA (tail-anchored) proteins, Get-3, Get-4, *Neurospora crassa*, *Aspergillus fumigatus*

## Abstract

Azole antifungals are widely used to control fungal infection, yet resistance mechanisms beyond drug efflux and target modification remain insufficiently understood. Here, we identified the Guided Entry of Tail-anchored proteins (GET) pathway as a conserved regulator of azole susceptibility in *Neurospora crassa* and *Aspergillus fumigatus*. Deletion or dysfunction of the core GET components Get-3 or Get-4 confers resistance to multiple azoles without impairing hyphal growth or sporulation. Mechanistically, GET deficiency neither reduced intracellular accumulation nor altered drug efflux and target expression or total ergosterol levels. Instead, GET-deficient strains displayed significantly reduced accumulation of the toxic sterol intermediate 14α-methyl-3,6-diol under azole stress, a change that may limit azole-induced sterol toxicity and contribute to reduced azole susceptibility. Proximity labeling and functional validation indicated that GET deficiency perturbs the trafficking of multiple azole-resistance-associated proteins, including the key tail-anchored protein transporter Emp-47 and mitochondrial Cox subunits Cox-4 and Cox-15, which deletion enhanced azole resistance. Transcriptomic and functional analyses further revealed that GET disruption altered the expression of some azole resistance-associated genes, which involve in membrane transport, metabolism, and cell wall organization, independent of canonical stress pathways. In this study, 19 previously uncharacterized genes modulating azole susceptibility are uncovered. Collectively, our findings identify the GET pathway as a non-classical regulator of azole susceptibility associated with protein-trafficking defects and metabolic adaptation in filamentous fungi.

**IMPORTANCE:**

Understanding non-canonical mechanisms of azole resistance is crucial given the rising failure of antifungal therapies. Here, we identify the GET pathway as an evolutionarily conserved regulator of azole susceptibility in both a model fungus and the major pathogen *Aspergillus fumigatus*. We uncover 19 previously unknown resistance-modulating genes and reveal a conceptually distinct mechanism whereby GET disruption confers resistance independently of classical efflux pumps or target alterations. Instead, GET dysfunction impairs trafficking of Emp-47 and Cox subunits, reduces toxic sterol intermediate accumulation, and alters resistance-associated gene expression. These findings establish protein trafficking as a new layer of stress adaptation and provide a mechanistic framework for further investigation of fungal azole susceptibility.

## INTRODUCTION

Fungal infections pose a significant and often underestimated global health threat, contributing to an estimated over 1 billion infections and nearly 3.75 million deaths annually (1). Azoles, which inhibit ergosterol biosynthesis by directly binding to sterol 14α-demethylase, represent the most widely used class of antifungals in both clinical and agricultural settings (2). However, resistance to azoles has emerged among numerous fungal pathogens and has become a serious problem leading to therapy failure of fungal infections (3–5). Therefore, a deeper understanding of the molecular mechanisms underlying antifungal resistance is critical for the development of novel therapeutic strategies.

Conventionally, azole resistance has been attributed primarily to the drug target and efflux pumps, such as their overexpression, target gene mutations, and mutations in the transcription factors that regulate them (6–8). In *Candida albicans*, azole resistance frequently arises from point mutations in the 14α-demethylase encoding gene *erg11*, leading to amino acid substitutions that reduce binding affinity to azoles (9, 10). Also in *Aspergillus fumigatus*, resistance commonly involves mutations in Cyp51A, the ortholog of Erg11 (11), or post-translational degradation of Cyp51A (12). Mutations causing over-expression of azole efflux pumps serve as another mechanism for fungal azole resistance, and these mutations are also frequently detected in azole-resistant isolates (13). Alterations in azole sensitivity are caused by deletions or mutations in transcription factors that regulate drug targets or efflux pumps. Key examples include Pdr1/3p (14, 15) and Upc2p (16) in yeast, Tac1p (17) and Upc2p (18) in *C. albicans*, as well as SrbA (19), AtrR (20), SltA (21), ZfpA (22), HapX (23), HapE (24, 25), and NctA (26) in *A. fumigatus*. Furthermore, transcription factors identified in *Neurospora crassa*, such as CCG-8 (27), ADS-4 (28), CSP-1 (29), and Crf4-3 (30), have been shown to play conserved roles, with their functional homologs characterized in *A. fumigatus* or *Fusarium verticillioides*. In addition, aneuploidy also confers azole resistance in *A. flavus* and *A. fumigatus* (31–33), which may be attributable to segmental duplications of regions encoding the azole target genes *cyp51A* and *cyp51B* in azole-resistant clinical isolates of *A. fumigatus* (32).

Beyond canonical resistance mechanisms, fungi employ several other well-established strategies to circumvent the inhibitory effects of azoles. For instance, the molecular chaperone Hsp90 orchestrates key stress response pathways that govern antifungal resistance (34, 35). Alternatively, disruption of Erg3- or ERG5- mediated alternative sterol biosynthesis enables fungal cells to accumulate non-toxic sterol intermediates under azole stress (36, 37). Biofilm formation represents another major strategy, wherein embedded fungal cells can exhibit 10- to 1,000-fold increased azole resistance through multifaceted mechanisms, including impaired drug penetration, metabolic quiescence, and extracellular matrix-mediated drug sequestration (38, 39). Furthermore, helicase MSP-8 deficiency drives multidrug resistance by reducing drug accumulation, remodeling the cell wall, and decreasing intracellular ROS levels (40). Despite these advances, resistance mechanisms beyond drug efflux and target modification remain poorly characterized.

The Guided Entry of Tail-anchored proteins (GET) pathway, which mediates the correct targeting of tail-anchored proteins (TAs) to the endoplasmic reticulum, is a key component of the protein transport system (41, 42). This pathway is highly conserved across yeast, plants, and mammals. TA proteins defined by a single C-terminal transmembrane domain (TMD) (43) constitute 3–5% of the eukaryotic membrane proteins and mediate diverse cellular processes, including protein translocation, vesicular transport, apoptosis, and protein quality control (44, 45). Although the GET pathway has been implicated in diverse cellular processes ranging from protein homeostasis to organelle biogenesis (46, 47), its potential role in antifungal resistance remains entirely unexplored.

By testing the azole susceptibility of a limited set of *N. crassa* deletion mutants, followed by genetic validation, we identified the GET pathway as a critical regulator of azole susceptibility in fungi. Deletion of *get-3* or *get-4*, which encodes two core components of the GET pathway, resulted in reduced fungal susceptibility to multiple azoles. Our findings implicate GET-dependent protein transport in the regulation of fungal responses to azole stress, adding a previously underappreciated dimension to established resistance mechanisms, such as drug efflux and target modification. Together, these findings broaden the current mechanistic framework of fungal azole resistance by highlighting protein transport as an additional regulatory layer in the cellular response to azole stress.

## MATERIALS AND METHODS

### Strains and culture conditions

The *Neurospora crassa* wild-type strain (FGSC#4200), *get-3*^ko^ (FGSC#12320), and *get-4*^ko^ (FGSC#19388) were obtained from Fungal Genetic Stock Center (FGSC; https://www.fgsc.net/Neurospora/neurospora.html). Complementation strains were constructed by transforming the respective deletion mutants with plasmids pCB1532-*get-3* and pCB1532-*get-4*. A *get-3* complementation strain with N-terminal Myc and TurboID tags was generated using plasmid pCB1532-Myc-TurboID-*get-3*. Strains were routinely cultured on Vogel’s medium (supplemented with 2% (wt/vol) sucrose for slants or glucose for plates) solidified with 1.5% agar. For mycelial cultivation, strains were incubated in liquid Vogel’s medium supplemented with 2% (wt/vol) glucose. Sorbose plates (Vogel’s medium with 20 g/L sorbose, 0.5 g/L fructose, and 0.5 g/L glucose) were used to screen transformants. All *Neurospora* cultures were incubated at 28°C.

The *Aspergillus fumigatus* reference strain (Af293, FGSC#A1100) and the auxotrophic strain (CEA17) were kindly provided by Prof. Cheng Jin (Institute of Microbiology, Chinese Academy of Sciences). The *Afget3* deletion strain was constructed using a knockout fragment containing *Afget3* upstream and downstream flanking regions and the pyrG encoding region. After protoplast transformation, transformants were screened on minimal medium (1 g/L sodium glutamate, 10% glucose, 1× basic salt solution, pH 6.8). All strains were maintained on complete medium (2% [wt/vol] glucose, 0.5% [wt/vol] yeast extract, and 1× YG trace elements, 1.5% agar). For drug susceptibility assays and growth comparisons involving the CEA17 parental strain and the *Afget3*^ko^ mutant, the medium was supplemented with 5 mM uridine and 10 mM uracil (UU). Although the *Afget3* mutant (which carries the pyrG marker) can grow without UU, the parental CEA17 strain is a pyrG auxotroph and requires UU for growth. To ensure identical culture conditions for a meaningful comparison, both strains were cultured in the presence of UU in all experiments. All *Aspergillus* cultures were grown at 37°C. Details of all strains are summarized in Table S1.

### Drug susceptibility test

Ketoconazole (KTC; Sigma), voriconazole (Vori; Sigma), and itraconazole (ITA; Merck Millipore) were dissolved in dimethyl sulfoxide (DMSO), followed by filtration sterilization. The drug solutions were added to medium when needed. The final solvent concentration in media was below 0.25% (vol/vol).

For radial growth assays, 2 μL of conidial suspension (2 × 10^6^ conidia/mL) from 7-day-old cultures was inoculated onto medium plates with or without azoles. Plates were incubated at 28°C in the dark, and colony diameters were measured after a defined incubation period. All experiments were independently repeated in triplicate.

To further evaluate antifungal susceptibility, minimum inhibitory concentrations (MICs) of KTC, Vori, and ITA were determined using broth microdilution assays. For *A. fumigatus*, MIC testing was performed according to the Clinical and Laboratory Standards Institute (CLSI) M38-A2 guideline. Conidial suspensions were prepared in RPMI 1640 medium, inoculated into 96-well microtiter plates containing twofold serial dilutions of antifungal drugs, and incubated at 37°C for 48 h. For *N. crassa*, broth microdilution assays were performed using the same experimental principle, except that plates were incubated at 28°C for 48 h. MIC values were defined as the lowest concentration resulting in complete inhibition of visible growth.

### Protein extraction and western blotting

Protein extraction was conducted as previously described, with some modifications. Fifty microliters from a conidial suspension (2 × 10^8^ conidia/mL) was added to 50 mL of liquid Vogel’s medium and incubated at 28°C with shaking at 200 rpm for 24 h. For TurboID-based proximity labeling, 4 μM biotin was added during cultivation. Harvested mycelia were flash-frozen in liquid nitrogen, ground into powder, and lysed in extraction buffer (50 mM HEPES [pH 7.4], 137 mM NaCl, 10% glycerol) supplemented with protease, phosphatase, and deacetylase inhibitors. Lysates were clarified by centrifugation (4°C, 12,000 rpm, 30 min), and protein concentrations were quantified using a BCA assay (Vazyme, China).

For SDS-PAGE, 30 μg protein per lane was loaded. After electrophoresis, proteins were transferred to PVDF membranes and probed with high-sensitivity streptavidin-HRP (Thermo Scientific, USA) to detect biotinylated proteins. Immunoreactive bands were visualized using an enhanced chemiluminescence (ECL) substrate (Easybio, China) and imaged with a chemiluminescence imaging system (Tanon, China).

### Streptavidin pull-down and LC–MS/MS analysis

Biotinylated proteins were enriched using Pierce MS-Compatible Magnetic IP Kit according to the manufacturer’s instructions. In brief, 25 µL of beads were equilibrated with cell lysis buffer. Then 1 mL of protein extract was incubated with the beads at room temperature for 1 h on a vertical mixer. Unbound proteins were removed, and beads were sequentially washed with wash buffer A and B. Biotinylated proteins were eluted using elution buffer and incubated for 10 min with shaking. Eluates were subjected to either liquid chromatography–mass spectrometry/MS (LC–MS/MS) or western blotting.

LC–MS/MS was performed on the TripleTOF 5600 (SCIEX, USA). The MS raw data for each sample were combined and searched using MaxQuant (v1.5.3.17) for protein identification and quantitation.

### RNA extraction and quantitative real-time RT-PCR (qRT-PCR)

Samples for RNA extraction were prepared as those for protein extraction. To examine drug-induced gene expression changes, KTC (2 μg/mL) was added after 12 h of culture, followed by an additional 12 h of incubation. Total RNA was extracted according to the standard TRIzol protocol (Invitrogen, USA). The cDNAs were synthesized from total RNA (1 μg) using a reverse transcription kit (Vazyme, China). The qRT-PCR analysis was performed on a CFX96 multicolor real-time PCR detection system (Bio-Rad, Hercules, CA, USA) with SYBR Green detection according to the manufacturer’s instructions. Gene-specific primers were designed using the online tools, PrimerQuest (https://sg.idtdna.com/pages/tools/primerquest).

### RNA-seq

RNA-seq was performed separately for the WT, *get-3*^ko^, and *get-4*^ko^ strains and for the WT and *emp-47*^ko^ strains with or without KTC treatment. Each condition included three biological replicates. Total RNA was extracted as described above and sequenced on a BGISEQ-500 platform.

For the bioinformatics analysis pipeline, raw sequencing reads were first filtered using SOAPnuke (v1.4.0) and Trimmomatic (v0.36) to remove low-quality reads, adapter contamination, and reads with a high content of ambiguous bases (N). The resulting clean reads were aligned to the *N. crassa* OR74A reference genome using HISAT2 (v2.1.0). For expression quantification, the clean reads were also aligned to the reference transcriptome using Bowtie2 (v2.2.5), and gene-level count estimates and FPKM values were obtained using RSEM (v1.2.8). Within each RNA-seq data set, genes with FPKM <10 in every sample were excluded. Differential expression was analyzed using DESeq2 based on the RSEM-derived count matrix. Genes with a Benjamini–Hochberg-adjusted *P* value <0.05 and |log2FC| ≥1 were considered differentially expressed.

### HPLC–MS analysis of sterol composition and drug accumulation

Samples for sterol profiling were prepared as described for protein extraction. Mycelial samples were heat-dried at 80°C, and then ground into fine powder. Approximately 10 mg of mycelial powder was used for sterol and KTC extraction in Agilent vials (2 mL). Quantification was performed using high-performance liquid chromatography–mass spectrometry (HPLC–MS) with ergosterol and KTC standards following established protocols (37).

### Bioinformatic analysis of tail-anchored (TA) proteins

Putative TA proteins in *N. crassa* were initially identified via FungiDB (https://fungidb.org) by filtering for proteins with a single transmembrane domain and lacking a signal peptide. The position of the transmembrane domain for each protein was then further analyzed using SMART (http://smart.embl-heidelberg.de/), resulting in 98 predicted TA proteins (Table S2). Protein–protein interactions were mapped using STRING (https://cn.string-db.org/).

## RESULTS

### Disruption of *get-3* or *get-4* reduces fungal susceptibility to antifungal azoles

**Fig 1:**
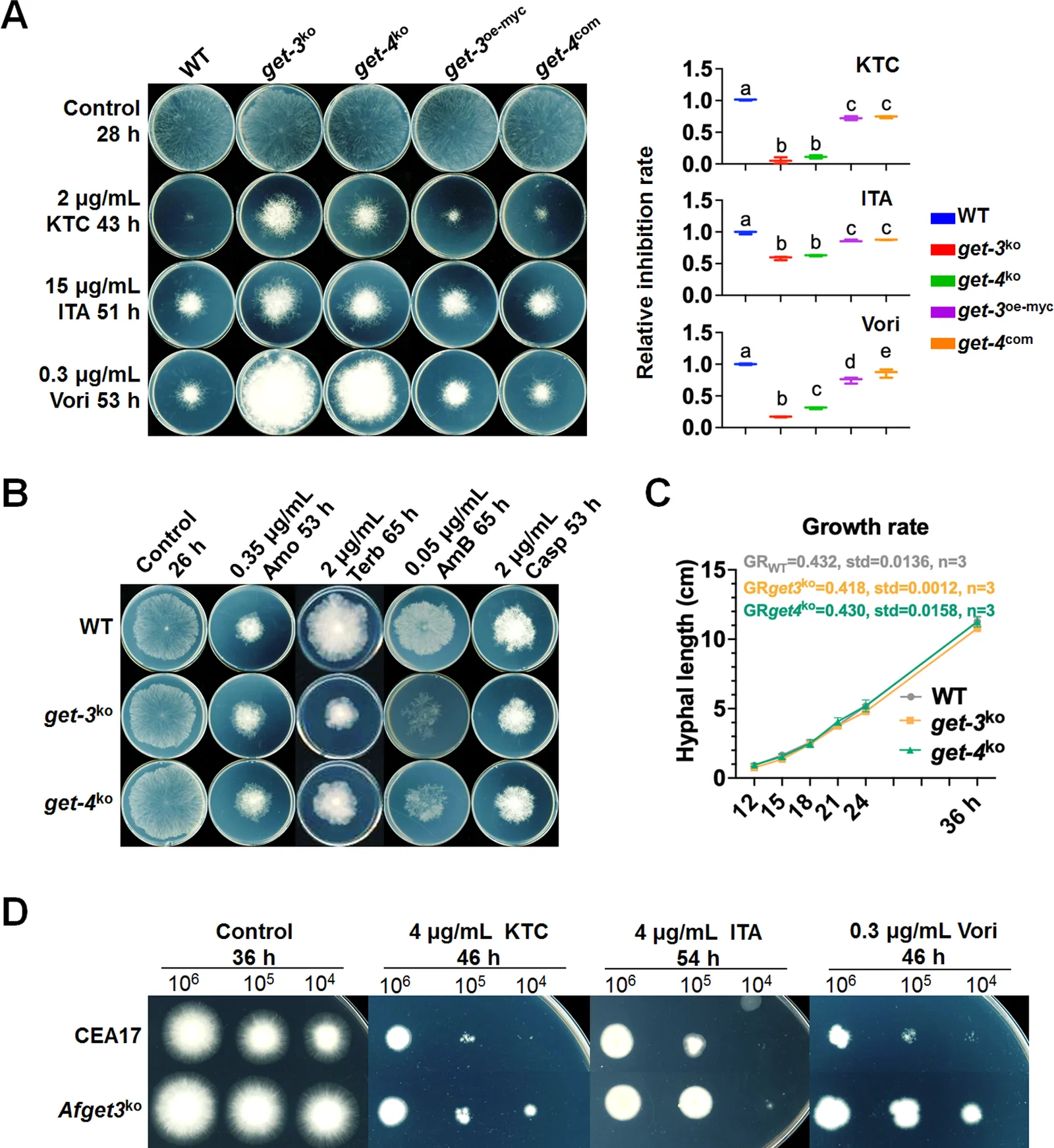
Disruption of the GET pathway confers azole resistance in *Neurospora crassa* and *Aspergillus fumigatus*. (**A**) Drug susceptibility assay of indicated *N. crassa* strains: the wild-type (WT) strain, the *get-3* knockout mutant (*get-3*^ko^), the *get-4* knockout mutant (*get-4*^ko^), the *get-3* complemented strain (*get-3*^oe-myc^), and the *get-4* complemented strain (*get-4*^com^). Left panel: growth on plates without drug or supplemented with ketoconazole (KTC), itraconazole (ITA), or voriconazole (Vori). Right panel: relative inhibition rates under KTC, ITA, and Vori. (**B**) Susceptibility of WT, *get-3*^ko^, and *get-4*^ko^ strains to different classes of clinical antifungals. Drugs tested include caspofungin (Casp), amphotericin B (AmB), amorolfine (Amo), and terbinafine (Terb). (**C**) Growth rates of the WT, *get-3*^ko^, and *get-4*^ko^ strains assessed by measuring colony diameter over time (12–36 h). Data are presented as mean ± SD (*n* = 3). (**D**) Azole susceptibility of *A. fumigatus* CEA17 parental strain and the *Afget3* knockout (*Afget3*^ko^) strain. For drug susceptibility assays comparing CEA17 and *Afget3*^ko^, the medium was supplemented with 5 mM uridine and 10 mM uracil (UU) to support the growth of the pyrG auxotrophic parental strain while maintaining identical culture conditions for the mutant. All experiments were performed with three biological replicates.

In the course of testing a limited set of *N. crassa* deletion mutants, we noticed that loss of *get-4* reduced azole susceptibility. Given the functional coupling of Get-3 and Get-4, we further examined a *get-3* knockout mutant and found a similar phenotype. Deletion of either *get-3* or *get-4* resulted in enhanced growth in the presence of ketoconazole (KTC), itraconazole (ITA), and voriconazole (Vori). Specifically, while the wild-type (WT) strain was significantly inhibited by KTC with an inhibition rate of 93.75% ± 0.32%, *get-3*^ko^ and *get-4*^ko^ strains only exhibited inhibition rates of 59.55% ± 1.74% and 61.59% ± 0.88%, respectively. Reduced susceptibility was also observed against ITA (WT: 77.20% ± 0.81%; *get-3*^ko^: 61.99% ± 1.11%; *get-4*^ko^: 63.55% ± 0.41%) and Vori (WT: 82.09% ± 0.45%; *get-3*^ko^: 50.63% ± 0.26%; *get-4*^ko^: 55.90% ± 0.59%) (Fig. 1A).

To confirm that the observed resistance phenotypes were directly caused by the deletion of *get-3* or *get-4*, we constructed and analyzed genetic complementation strains. Reintroduction of the native *get-3* and *get-4* genes into the respective null mutants restored azole susceptibility to the level of the wild-type strain (Fig. 1A). These results demonstrate that both *get-3* and *get-4* are required for maintaining azole susceptibility in *N. crassa*.

To determine whether the role of the GET pathway extends beyond azole resistance, we assessed the susceptibility of the *get-3*^ko^ and *get-4*^ko^ strains to other clinical antifungals belonging to different classes, including amphotericin B (AmB, polyenes), amorolfine (Amo, morpholine), terbinafine (Terb, allylamine), and caspofungin (Casp, echinocandins). As shown in Fig. 1B, while both mutants exhibited increased sensitivity to AmB and Terb, no significant changes were found in response to Casp or Amo. These findings suggest that the GET pathway does not confer broad-spectrum antifungal resistance, but preferentially affects azole susceptibility.

Notably, *get-3*^ko^ and *get-4*^ko^ strains showed no significant defects in hyphal growth (Fig. 1A and C) and sporulation (Fig. S1). This indicates that their reduced azole susceptibility is not a consequence of generalized growth or developmental impairment, but rather points to a specific role for the GET pathway in the antifungal response.

Given the evolutionary conservation of the GET pathway, we extended our investigation to the clinically important pathogen *Aspergillus fumigatus*. We generated a deletion mutant of the *Afget3* ortholog (*Afget3*^ko^, AFUA_3G11350) in the CEA17 background. For all growth and drug susceptibility assays comparing the CEA17 parental strain and the *Afget3*^ko^ mutant, the medium was supplemented with 5 mM uridine and 10 mM uracil (UU) to support the growth of the pyrG auxotrophic parental strain while maintaining identical culture conditions. Consistent with the findings in *N. crassa*, deletion of *Afget3* in *A. fumigatus* resulted in enhanced growth in the presence of KTC, Vori, and ITA compared to the parental strain (Fig. 1D).

Furthermore, a clinically derived strain (*Afget4*^E46A^) with a point mutation in Get-4 (AFUA_6G05050; E46A), which was kindly provided by Prof. Wenxia Fang (Institute of Biological Sciences and Technology Guangxi Academy of Sciences), was also analyzed. It is noteworthy that this clinical isolate also exhibited enhanced growth on azole-containing medium (Fig. S1B). Importantly, this finding does not demonstrate causality. While this observation is intriguing and suggests that impaired Get-4 function may be associated with resistance in natural settings, the complex genetic background of a clinical isolate means that this phenotype cannot be definitively attributed to the Get-4 (E46A) mutation alone.

Because enhanced growth on azole-containing solid medium does not necessarily indicate an elevated MIC, we further determined the MIC values of KTC, Vori, and ITA using broth microdilution assays according to the CLSI M38-A2 method. In *A. fumigatus*, both the *Afget-3^ko^* mutant and the *Afget-4^E46A^* mutant exhibited twofold increases in the MICs of KTC (2.0 to 4.0 μg/mL), Vori (0.125 to 0.25 μg/mL), and ITA (0.25 to 0.5 μg/mL) compared with their reference strains, CEA17 and Af293. In *N. crassa*, deletion of either *get-3* or *get-4* similarly resulted in twofold increases in the MICs of all three azoles, from 1.0 to 2.0 μg/mL for KTC, 0.5 to 1.0 μg/mL for Vori, and 2.0 to 4.0 μg/mL for ITA. Together, these results demonstrate that disruption of the GET pathway consistently reduces azole susceptibility across *A. fumigatus* and *N. crassa*, with elevated MICs observed for multiple azoles (Table S1).

Nevertheless, these results support the conclusion that the GET pathway is an important regulator of azole susceptibility in *N. crassa* and *A. fumigatus*, underscoring its conserved role across filamentous fungi.

### GET pathway disruption is associated with sterol metabolic remodeling rather than reduced drug accumulation or target overexpression under azole stress

**Fig 2:**
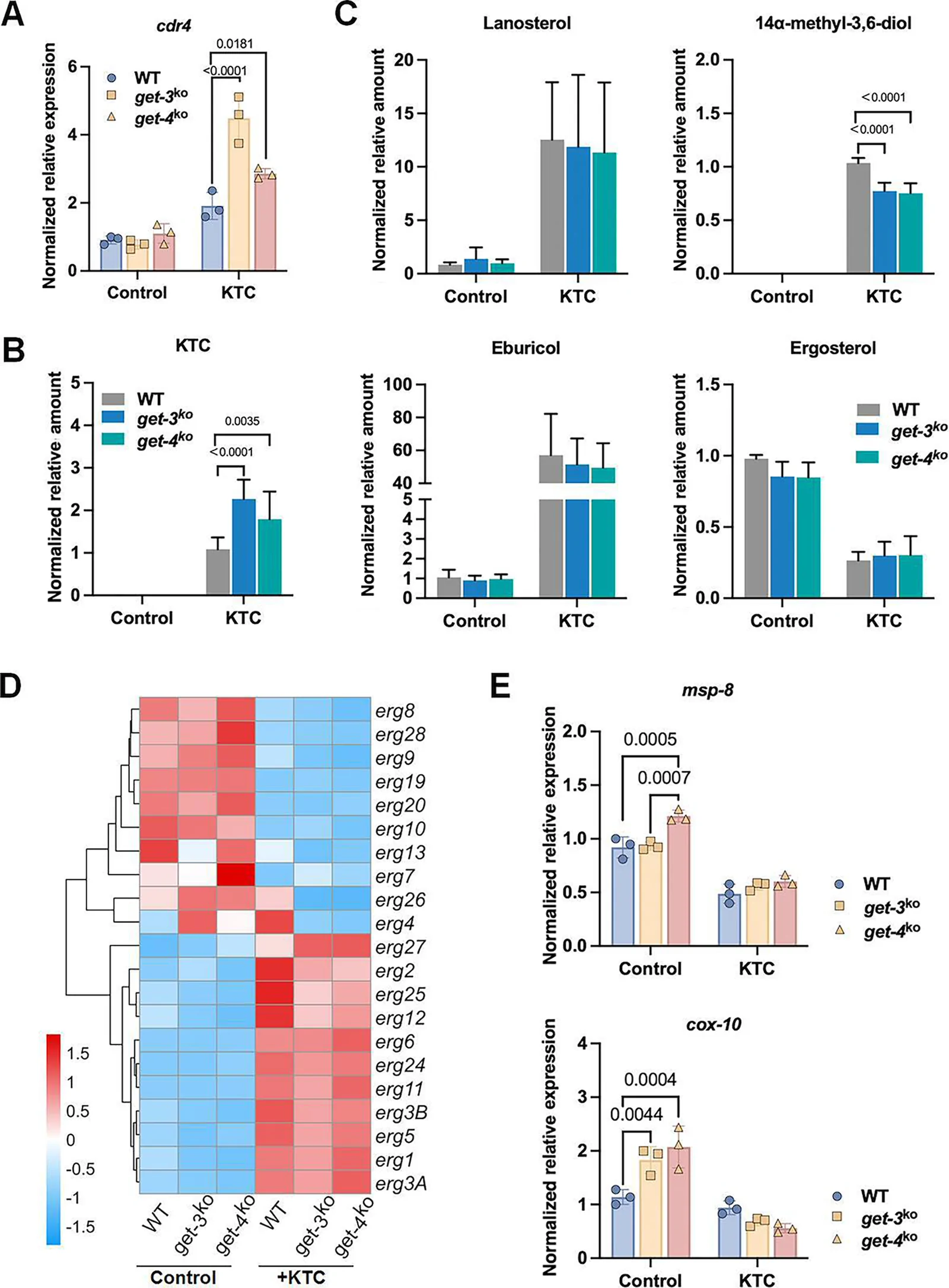
GET pathway disruption is associated with sterol metabolic remodeling without reduced intracellular drug accumulation or azole-target overexpression under azole stress. (**A**) Normalized relative expression levels of the major efflux pump gene *cdr4* in WT, *get-3*^ko^, and *get-4*^ko^ strains with or without KTC (2 μg/mL) treatment. Data are normalized to the untreated WT control (**B**) Intracellular accumulation of KTC in WT, *get-3*^ko^, and *get-4*^ko^ strains under KTC stress (*n* = 6). (**C**) Heatmap showing expression profiles of genes involved in ergosterol biosynthesis across the indicated strains. (**D**) LC-MS quantification of ergosterol and key intermediates (*n* = 6). (**E**) Normalized relative expression levels of *msp-8* and *cox-10* genes in WT, *get-3*^ko^, and *get-4*^ko^ strains with or without KTC (2 μg/mL) treatment. Data are normalized to the untreated WT control. All data represent mean ± SD. Statistical significance was determined by two-way ANOVA.

To investigate cellular changes associated with reduced azole susceptibility following GET pathway disruption, we first assessed drug efflux and target expression. Deletion of *get-3* or *get-4* did not alter the basal transcription level of the major efflux pump gene *cdr4*; however, upon KTC treatment, both *get-3*^ko^ and *get-4*^ko^ strains exhibited enhanced *cdr4* induction (2.3- and 1.5-fold higher than WT, respectively; *P* < 0.05; Fig. 2A). Despite the elevated transcription of *cdr4*, the intracellular accumulation of KTC was significantly higher in both knockout strains than in WT (Fig. 2B), indicating that increased efflux pump expression did not translate into enhanced drug clearance.

We further analyzed the expression of *erg11*, the target of KTC, and genes involved in the ergosterol biosynthesis pathway. In *get-3*^ko^ and *get-4*^ko^ mutants, the transcriptional levels of *erg11* remained unchanged compared with the wild-type strain (Fig. 2C). Additionally, no significant differences were observed in the expression of most ergosterol biosynthesis genes between mutants and the wild type, either in the absence or presence of KTC treatment (|log_2_FC| < 1) (Fig. 2C; Table S6). Similarly, the total ergosterol content and the levels of key biosynthetic intermediates (lanosterol and eburicol) showed no notable alterations under KTC-treated or untreated conditions (Fig. 2D). Together, these results demonstrate that although intracellular azole accumulation is elevated in *get-3*^ko^ and *get-4*^ko^ mutants, this does not effectively translate into inhibition of the ergosterol biosynthesis pathway. Together, these results indicate that the reduced azole susceptibility of GET mutants is unlikely to be explained by enhanced drug efflux or increased expression of the azole target.

Most notably, LC–MS analysis revealed a marked reduction in the accumulation of 14α-methyl-3,6-diol—a toxic sterol intermediate—by 25.4 and 27.4% in *get-3*^ko^ and *get-4*^ko^ strains, respectively, compared to WT (*P* < 0.001; Fig. 2D). Under treatment with KTC, late-stage sterol biosynthetic genes, including *erg26* and *erg4*, exhibited a slight downregulation (Fig. 2C). Previous studies have demonstrated that accumulation of 14α-methyl-3,6-diol can alter membrane biophysical properties by reducing rigidity and phase heterogeneity, thereby conferring fluconazole hyper-resistance in *Saccharomyces cerevisiae* and azole resistance in *Candida albicans* (48, 49). The reduced accumulation of this deleterious sterol metabolite in GET-deficient mutants suggests a metabolic strategy that preserves membrane integrity by circumventing azole-induced sterol toxicity. Given previous findings that MSP-8 and Cox-10 mediate drug susceptibility through non-canonical mechanisms (40, 50), we, thus, investigated whether their expression is regulated by the GET pathway. In the absence of KTC, expression of *msp-8* and *cox-10* was elevated in both *get-3*^ko^ and *get-4*^ko^ strains compared with the wild type (Fig. 2E). However, under KTC treatment, transcript levels of the two genes showed no significant difference between mutants and the wild-type strain (Fig. 2E). These results indicate that while GET pathway disruption may mildly up-regulate *msp-8* and *cox-10* under basal conditions, this transcriptional response is not sustained or amplified under drug stress. Together with the unchanged ergosterol pathway gene expression, these data further support that GET-mediated azole resistance operates independently of previously described nontraditional resistance mechanisms linked to *msp-8* or *cox-10*.

### The GET pathway interacts with resistance-associated tail-anchored proteins

**Fig 3:**
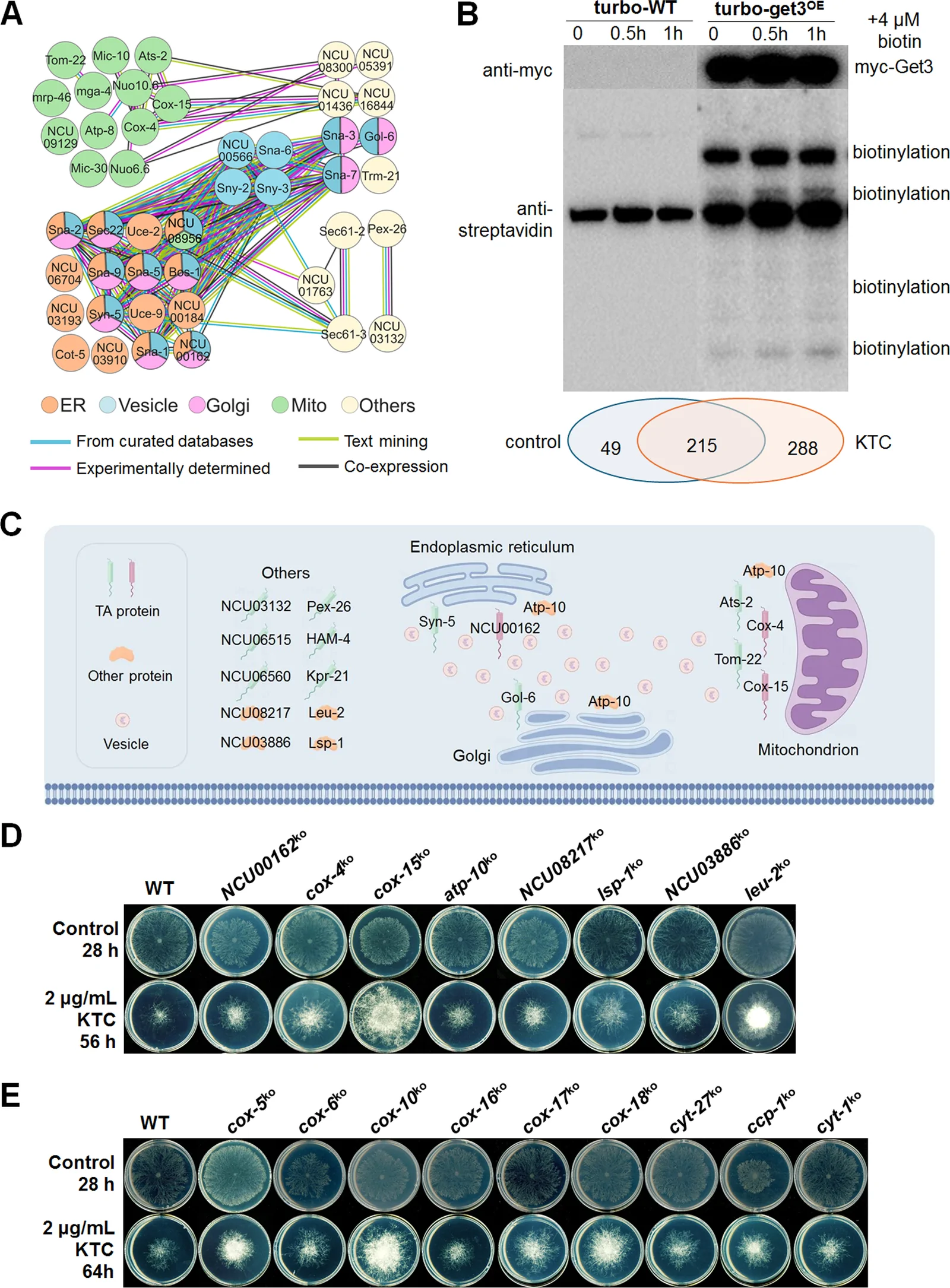
The GET pathway regulates azole resistance through mediating protein trafficking modulation. (**A**) Classification of 98 candidate TA proteins based on subcellular localization prediction and functional annotation. Proteins were categorized into five major groups: endoplasmic reticulum (ER)-localized (16 proteins, e.g., Sna-1 and Sna-2), Golgi apparatus-targeted (11 proteins, e.g., Gol-6 and Sna-3), mitochondrial (13 proteins, e.g., Cox-4 and Cox-15), vesicular transport-associated (16 proteins, e.g., Sny-2 and Sny-3), and others (43 proteins, some proteins without interaction relationships are not shown). The protein-protein interaction network (STRING database) highlights two functionally coherent modules: ER-Golgi vesicle trafficking module (bottom left corner, containing SNARE complex components) and mitochondrial electron transport chain module (top left corner, comprising Cox family proteins). The complete data set is available in Table S2. (**B**) Streptavidin western blot confirming specific biotinylated proteins in the TurboID-Get-3 strain (top panel). Venn diagram comparing proteins identified by proximity labeling under untreated and KTC-treated conditions (bottom panel). (**C**) TA and client proteins of the GET pathway identified via multi-method integration (schematic created with Figdraw; https://www.figdraw.com/#/). (**D**) Drug susceptibility profiling of Get-3-interacting protein deletion mutants against 2 µg/mL KTC. (**E**) Drug susceptibility profiling of Cox family protein deletion mutants against 2 µg/mL KTC. All experiments were performed with at least three independent replicates.

Given the established role of the GET pathway in mediating the post-translational insertion of tail-anchored (TA) proteins into membranes, we hypothesized that its contribution to azole resistance may be exerted through regulating specific TA proteins. To explore this, we conducted a bioinformatic analysis to identify candidate TA proteins in *Neurospora crassa*. A total of 98 putative TA proteins were identified (Table S2). Among them, 16 were associated with the endoplasmic reticulum (e.g., Sna-1 and Sna-2), 15 with vesicular transport, 13 with mitochondria (e.g., Tom-22 and Cox-4), 11 with the Golgi apparatus, and 12 candidates were predicted to localize to multiple organelles (Fig. 3A; Table S2). Protein–protein interaction network analysis revealed a highly interconnected architecture among these candidates, implying coordinated regulation.

To identify TA proteins and other proteins that may directly interact with the GET pathway, we labeled Get-3 at the N‐terminus with TurboID and performed proximity labeling to identify Get-3-interacting proteins under both basal and KTC-treated conditions (51). Validation experiments confirmed that neither TurboID fusion nor biotin induction affected fungal growth or azole susceptibility (Fig. S2A to C), indicating that the labeling system did not interfere with Get-3 function in drug response. Biotin-induced proximity labeling was performed to capture proteins near Get-3 (Fig. 3B; Fig. S2C). The biotinylated proteins were subsequently enriched using streptavidin-coated beads and identified by mass spectrometry (MS). Under normal conditions, 264 Get-3-interacting proteins were identified, increasing to 503 under KTC exposure (Fig. 3B). Core GET pathway components (Get-1, Get-2, Get-4, Get-5), as well as previously reported client proteins in yeast (e.g., Erg1; Table S3) (52, 53), were reliably detected, validating the fidelity of the labeling system.

Among the predicted TA proteins, 13 high-confidence candidates were identified as direct Get-3 interactors (Table S3; Fig. 3C). Six of them—NCU00162, Sny-5, Gol-6, Ats-2, Kpr-21, and NCU06560—were detected in both untreated and KTC-treated conditions, suggesting their role as core clients of the GET pathway. Functional validation revealed that deletion of *NCU00162* (*NCU00162*^ko^) conferred increased resistance (Fig. 3D). NCU00162 shares significant sequence homology with Emp-47 proteins across multiple fungal species, including *A. fumigatus*, *Madurella mycetomatis*, *Podospora anserina*, and *Saccharomyces cerevisiae*. Emp-47 is an ER-Golgi cargo receptor involved in glycoprotein trafficking (54–57). It has been previously demonstrated in *A. fumigatus* that the deletion of Emp-47 increased resistance to KTC, Vori, ITA, and miconazole (54). Moreover, the *emp-47*^ko^ strains exhibit increased miconazole tolerance in *S. cerevisiae* (55), further supporting a conserved role for this protein in azole susceptibility.

Under KTC-induced stress, five additional predicted TA proteins—Cox-4, Cox-15, Tom-22, Ham-4, and NCU03132—were identified as Get-3 interactors (Table S3). Among them, deletion of *cox-4* and *cox-15* significantly enhanced azole resistance (Fig. 3D). Cox-4 and Cox-15 are components involved in the assembly and function of mitochondrial cytochrome c oxidase (complex IV), and dysfunction of complex IV has previously been associated with altered azole susceptibility in fungi (50, 58, 59). Consistent with this, we further examined additional complex IV components and found that deletion of multiple Cox subunits, including Cox-5, Cox-10, Cox-17, and Cox-18, similarly reduced susceptibility to KTC (Fig. 3E). These results extend previous observations in *A. fumigatus* that disruption of complex IV components, including Cox7c and Cox10, promotes azole resistance (58, 59). Our data reinforce the crucial role of mitochondria in regulating antifungal drug responses (50, 58–60) and suggest that the GET pathway is involved in trafficking mitochondrial resistance modulators.

Additionally, we identified several novel Get-3-interacting proteins. Among them, deletion of *atp-10*, *lsp-1*, *NCU03886*, *NCU08217*, and *leu-2* led to increased resistance to KTC (Fig. 3D; Table S3). These proteins are involved in diverse biological processes: Atp-10 encodes a mitochondrial membrane-associated ATPase (61). Lsp-1, a plasma membrane protein, forms the eisosome complex with Pil1 beneath the membrane compartment of Can1 and is crucial for sphingolipid signaling transduction (62–64); NCU03886 is predicted to encode a PH domain-containing protein, often involved in membrane localization and signaling (FungiDB). NCU08217 encodes SRP receptor beta subunit and is involved in protein targeting to the ER (FungiDB). Leu-2 is identified as 3-isopropylmalate dehydratase involved in leucine biosynthesis. The diverse cellular localizations and functions of these proteins suggest that GET-dependent protein targeting may be functionally connected to mitochondrial, plasma membrane, ER targeting, and metabolic processes, potentially through a broader regulatory network.

Collectively, these results suggest that the GET pathway orchestrates azole resistance by mediating the trafficking of multiple TA proteins—including Emp-47, Cox-4, and Cox-15—and coordinating a broader regulatory network encompassing vesicular transport, mitochondrial function, and membrane lipid homeostasis. This represents a novel, multifaceted mechanism of fungal azole resistance beyond conventional target or efflux-based paradigms.

### Loss of Emp-47 remodels basal cellular homeostasis associated with azole susceptibility

To investigate how disruption of the GET-associated cargo protein Emp-47 contributes to reduced ketoconazole susceptibility, we performed comparative transcriptomic analysis of WT and *emp-47*^ko^ strains with or without KTC treatment. After filtering out lowly expressed genes (FPKM <10), 5,848 genes were retained for subsequent analysis. Differentially expressed genes were identified using thresholds of *P* <0.05 and |log_2_FC| ≥1. Under normal growth conditions, deletion of emp-47 altered the expression of 1,222 genes, including 881 upregulated and 341 downregulated genes, whereas only 33 genes were differentially expressed under KTC treatment (18 upregulated and 15 downregulated) (Table S4). Thus, the transcriptional impact of *emp-47* deletion was substantially more extensive under basal conditions than during KTC treatment.

GO enrichment analysis showed that genes upregulated in the *emp-47*^ko^ strain under control conditions were enriched in processes associated with translation and ribosome biogenesis, mitochondrial and energy metabolism, fatty acid β-oxidation, oxidative stress and redox homeostasis, and protein folding (Fig. S3E; Table S4). Downregulated genes were associated with transcriptional regulation, fungal cell wall organization, and cell surface-related components, including the plasma membrane, cell tip cortex, extracellular region, and fungal cell wall (Fig. S3E; Table S4). These results indicate that loss of Emp-47 is associated with extensive remodeling of basal cellular functions, particularly protein synthesis, mitochondrial and energy metabolism, and cell surface-associated processes.

We next examined representative genes involved in ROS responses, HSP90 signaling, calcium signaling, and detoxification. KTC treatment did not induce broad transcriptional activation of ROS-, HSP90-, or calcium signaling-related genes in the *emp-47*^ko^ strain (Fig. S3A–C; Table S5). Among detoxification-related genes, *cdr4* and *NCU08390* were downregulated, whereas the ABC transporter gene *NCU03776* was upregulated in the *emp-47*^ko^ strain relative to WT under KTC treatment (Fig. S3D; Table S5). Thus, the reduced KTC susceptibility of the *emp-47*^ko^ strain was not accompanied by coordinated transcriptional activation of these canonical antifungal stress-response pathways.

Comparison of KTC-responsive genes in WT with genes differentially expressed between *emp-47*^ko^ and WT under KTC treatment identified eight candidate genes. *NCU08680*, *NCU02071*, and *NCU09235* were induced by KTC in WT and further upregulated in *emp-47*^ko^; *NCU02030* was specifically induced in *emp-47*^ko^; and *NCU05752*, *NCU08407*, *NCU02895*, and *NCU00304* showed stronger repression in *emp-47*^ko^ than in WT (Fig. S3F; Table S4). Altered expression of these genes may be associated with the reduced KTC susceptibility of the *emp-47*^ko^ strain.

We further examined sterol metabolism and intracellular drug accumulation. KTC treatment caused ergosterol depletion and accumulation of lanosterol, eburicol, and 14α-methyl-3,6-diol in both WT and *emp-47*^ko^ strains, with no significant differences between the two strains under either control or KTC-treated conditions (Fig. S4B and E). Consistently, emp-47 deletion did not significantly alter *erg11* transcript levels (Fig. S4A). Moreover, although *cdr4* expression was decreased in *emp-47*^ko^ under KTC treatment, intracellular drug accumulation was not significantly reduced compared with WT (Fig. S4C and D). Together, these results suggest that the reduced KTC susceptibility of *emp-47*^ko^ is not primarily associated with increased *erg11* expression, transcriptional upregulation of *cdr4*, altered sterol abundance, or reduced intracellular drug accumulation, but is accompanied by broader remodeling of basal cellular homeostasis.

### Disruption of the GET pathway alters the expression of azole resistance-associated genes

**Fig 4:**
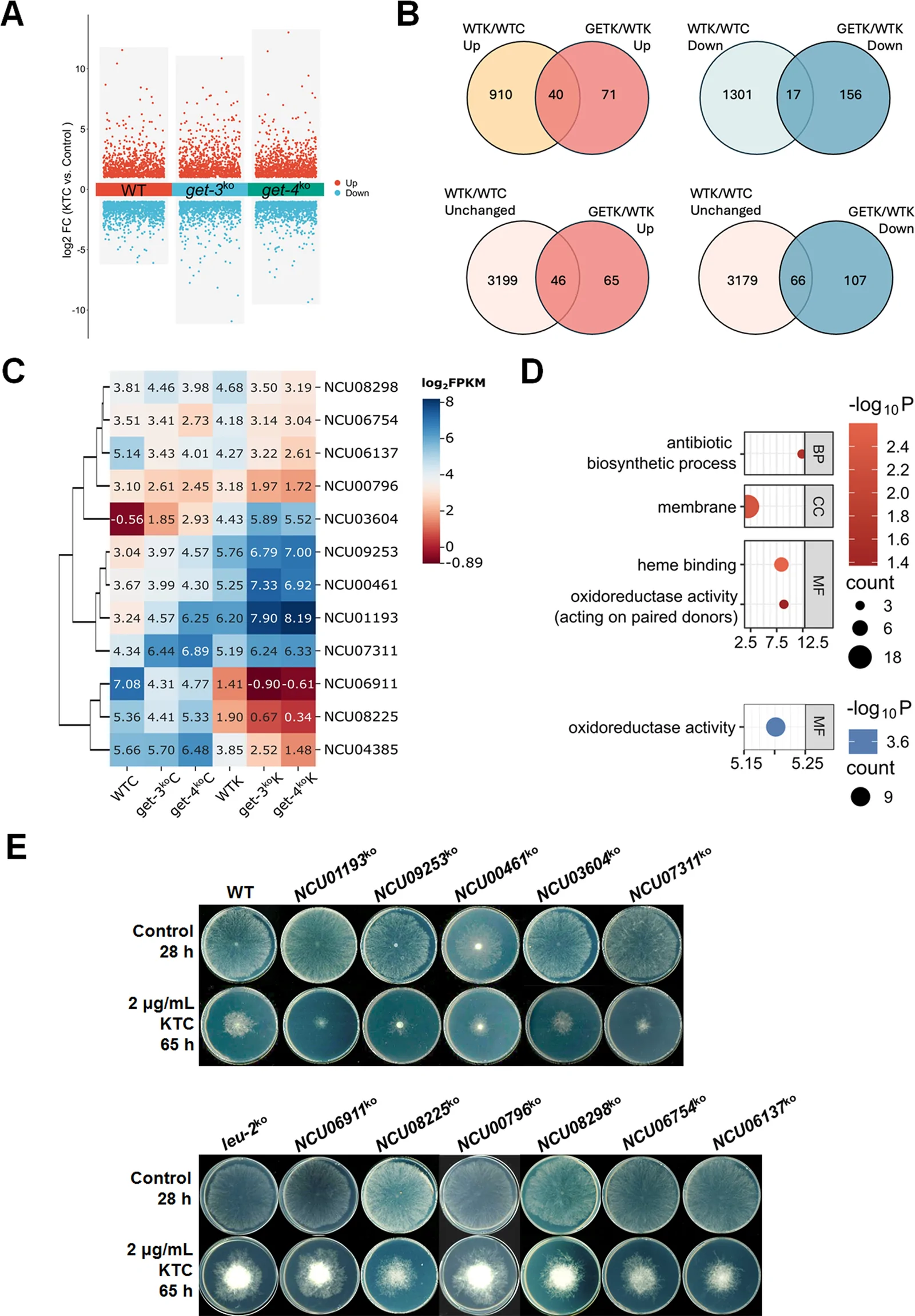
Disruption of the GET pathway impairs the expression of azole resistance-associated genes. (**A**) Volcano plots showing differentially expressed genes after ketoconazole treatment compared to untreated control in WT, *get-3*^ko^, and *get-4*^ko^ strains. Significantly upregulated and downregulated genes (|log_2_FC| ≥  1, *P*  < 0.05) are colored red and blue, respectively. (**B**) Venn diagram representing four gene sets: (i) genes upregulated in WT that show further enhanced upregulation (≥ 2 fold) in *get-3*^ko^ and *get-4*^ko^ mutants (*n*  =  40); (ii) genes specifically upregulated in the knockout strains but unchanged in WT (*n*  =  46); (iii) genes downregulated in WT with more pronounced downregulation (≥ 2 fold lower) in the knockouts (*n*  =  17); and (iv) genes specifically downregulated in the knockouts but unchanged in WT (*n*  =  66). (**C**) Heatmap of differentially expressed genes that contribute to azole resistance in GET-deficient backgrounds. (**D**) Gene Ontology (GO) enrichment analysis of upregulated (top) and downregulated (bottom) gene sets. Top panel: genes upregulated in WT and further enhanced in knockouts (*n*  =  40) plus genes specifically upregulated only in knockouts (*n*  =  46). Bottom panel: genes downregulated in WT and further reduced in knockouts (*n*  =  17) plus genes specifically downregulated only in knockouts (*n*  =  66). (**E**) KTC susceptibility of deletion mutants of the differentially expressed genes shown in (**C**) assessed at 2 µg/mL KTC.

To further elucidate the molecular mechanisms underlying GET pathway-mediated azole resistance, we performed comparative transcriptomic analysis of WT, *get-3*^ko^, and *get-4*^ko^ strains cultured in the presence or absence of KTC. A total of 5,522 transcripts exhibiting robust expression (FPKM ≥10 across all samples) were retained for downstream analyses. Upon KTC exposure, the WT strain exhibited differential expression (|log_2_FC| ≥ 1, *P* < 0.05) of 2,198 genes, including 1,011 upregulated and 1,187 downregulated transcripts (Fig. 4A; Table S6). In contrast, the *get-3*^ko^ and *get-4*^ko^ strains displayed distinct yet partially overlapping transcriptional responses, with 2,048 (1,036 upregulated, 1,012 downregulated) and 2,217 (1,097 upregulated, 1,120 downregulated) genes significantly altered, respectively (Fig. 4A; Table S6).

To determine whether established stress response systems contribute to GET-mediated azole resistance, we examined the expression of genes involved in ROS pathway (e.g., *sod1* and *cat1*; Fig. S3A; Table S7), the HSP90 pathway (e.g., *p23 *and *aha1*; Fig. S3B; Table S7), and the calcium-signaling pathway (e.g., *cna1* and *crz1*; Fig. S3C; Table S7), as well as detoxification genes (ABC/MFS transporters, e.g., *abc-8*; Fig. S3D; Table S7). However, the transcriptional levels of these core stress response genes remained largely unchanged (fold change <2) in GET mutants under KTC exposure relative to the WT strain (Fig. S3A-D; Table S7). These findings indicate that GET pathway-mediated resistance is independent of canonical stress signaling cascades typically associated with antifungal adaptation.

To identify functional contributors among GET-regulated transcripts, we assessed the azole susceptibility of knockout mutants corresponding to differentially expressed genes. Under KTC stress, genes involved in the azole response, including those encoding drug efflux pumps and target enzymes, exhibited altered expression patterns. Notably, 40 genes were upregulated in the WT and exhibited even stronger upregulation (≥2 fold) in both *get-3*^ko^ and *get-4*^ko^ mutants (Fig. 4B and C; Table S8). Among these, deletion of *NCU01193*, *NCU09253*, *NCU00461*, or *NCU03604* resulted in increased azole sensitivity (Fig. 4E), implicating their elevated expression as a key contributor to resistance in GET-deficient mutants. Specifically, *NCU01193* encodes a putative flavin-binding oxidoreductase; *NCU09253* encodes a DUF907-domain transmembrane protein implicated in cell wall biogenesis; *NCU00461* encodes the NAD^+^-specific glutamate dehydrogenase GDH-1 involved in nitrogen and glutamate metabolism; and *NCU03604* encodes a functionally uncharacterized protein (FungiDB and UniProt). Collectively, these genes are associated with primary metabolism and redox homeostasis, cell wall/membrane integrity, and yet-unresolved cellular functions.

Conversely, among genes downregulated in the WT strain under KTC treatment, 17 exhibited more pronounced repression in both *get-3*^ko^ and *get-4*^ko^ mutants (≥2 fold lower than WT; Fig. 4B and C; Table S8). Disruption of *NCU04385* (*leu-2*), *NCU06911*, or *NCU08225* (*trm-34*) conferred increased azole resistance (Fig. 4E), indicating that suppression of these genes contributes to GET-mediated resistance. These genes encode, respectively, 3-isopropylmalate dehydratase involved in leucine biosynthesis, a secreted hypothetical protein linked to oxidative stress, and a high-affinity nickel transporter.

In addition, we also identified 46 genes whose expression was not significantly altered in the WT strain under KTC treatment but was markedly upregulated in both *get-3*^ko^ and *get-4*^ko^ mutants (Fig. 4B and C; Table S8). Deletion of *NCU07311* enhanced azole sensitivity (Fig. 4B). NCU07311 encodes a MARVEL domain-containing protein, suggesting that specific high expression of *NCU07311* may contribute to resistance in GET-deficient backgrounds. Conversely, 66 genes showed no significant change in WT under KTC treatment yet were significantly downregulated in both mutants (Fig. 4B and C; Table S8). Deletion of *NCU06754* (*znf-1*), *NCU00796*, *NCU08298* (*pla-3*), and *NCU06137* (*cyp450-13*) enhanced azole resistance (Fig. 4B). The corresponding proteins include a CHY zinc finger domain-containing protein (NCU06754, Znf-1), a hydantoinase involved in glutathione metabolism (NCU00796), phospholipase-3 (Pla-3), and a cytochrome P450-like protein involved in antibiotic biosynthetic processes (NCU06137, Cyp450-13). This suggests that reduced expression of these genes may also contribute to resistance in GET-deficient backgrounds.

To delineate the transcriptional alterations induced by GET pathway dysfunction, we performed Gene Ontology (GO) enrichment analysis on the aforementioned gene sets, including genes upregulated in WT with further enhanced upregulation (≥2 fold) in *get-3*^ko^ and *get-4*^ko^ mutants (*n* = 40), genes specifically upregulated in the knockout strains but unchanged in WT (*n* = 46), genes downregulated in WT with more pronounced downregulation (≥2 fold lower) in the knockouts (*n* = 17), and genes specifically downregulated in the knockouts but unchanged in WT (*n* = 66) (Fig. 4B; Table S8).

Among upregulated gene sets, significant enrichment was observed for antibiotic biosynthetic process (fold enrichment = 12.35, *P* < 0.05), membrane (fold enrichment = 1.90, *P* < 0.05), heme binding (fold enrichment = 8.40, *P* < 0.05), and oxidoreductase activity (fold enrichment = 8.87, *P* < 0.05). Notably, genes specifically downregulated in *get-3*^ko^ and *get-4*^ko^ strains were also enriched for oxidoreductase activity (fold enrichment = 5.2, *P* < 0.05) (Fig. 4D). These findings suggest that GET pathway dysfunction disrupts membrane remodeling and is linked to oxidoreductase activity—processes known to critically influence antifungal susceptibility.

Taken together, our integrated transcriptomic and functional analyses demonstrate that the GET pathway exerts a regulatory control over a specific subset of genes essential for azole resistance. This regulatory network is distinct from canonical drug efflux systems and classical stress-response pathways, revealing a previously unrecognized layer of transcriptional regulation that contributes to the emergence of non-classical resistance phenotypes in filamentous fungi.

## DISCUSSION

The current understanding of antifungal resistance has been dominated by two major paradigms: drug target alteration and efflux pump-mediated resistance (65–68). However, emerging evidence suggests the existence of additional, mechanistically distinct resistance pathways that operate independently of these classical mechanisms (40, 69). The molecular basis of such non-canonical resistance remains largely elusive, emphasizing the necessity to systematically investigate novel resistance genes that may uncover resistance mechanisms beyond drug efflux and target modification and yield innovative therapeutic strategies. Our study identified the GET pathway as a central regulator of fungal azole susceptibility through distinct but interconnected mechanisms: remodeling alternative sterol metabolism to circumvent azole-induced toxicity, and coordinated regulation of protein transport including Emp-47, Cox subunits, etc. and resistance-related genes involved in membrane and metabolic remodeling. This represents a novel, multifaceted mechanism of fungal azole resistance beyond conventional target or efflux pump-mediated paradigms.

Our findings demonstrate that disruption of GET pathway specifically confers resistance to azoles while increasing susceptibility to terbinafine and amphotericin B. Supporting this, Farkas et al. revealed in yeast that the GET pathway regulates the targeting of the non-canonical client Erg1, disrupting sterol biosynthesis and consequently enhancing terbinafine sensitivity (53). Through proximity labeling, we similarly identified an interaction between Erg1 and Get-3. Notably, GET pathway deficiency led to reduced *erg1* expression (Table S6), suggesting that impaired GET function likely affects both Erg1 targeting and transcriptional regulation, thereby increasing terbinafine sensitivity. Remarkably, this differential susceptibility profile, azole tolerance alongside terbinafine/amphotericin B sensitivity, suggests that GET pathway status may have potential implications for antifungal drug selection.

Our findings suggest that GET pathway disruption is associated with remodeling of sterol metabolism rather than resistance driven by altered drug accumulation or azole-target overexpression. Under KTC-induced ergosterol suppression, GET-deficient mutants exhibited reduced accumulation of the toxic sterol intermediate 14α-methyl-3,6-diol, accompanied by modest downregulation of sterol biosynthesis genes, including *erg26* and *erg4* (Fig. 2C). Previous studies have established the relevance of reduced 14α-methyl-3,6-diol formation to azole resistance. In *Candida albicans*, loss of Erg3 function prevents the formation of this toxic sterol and confers high-level azole resistance (49) , while in *N. crassa*, deletion of *csp-1* reduced susceptibility to azoles on solid medium and was accompanied by decreased KTC-induced accumulation of 14α-methyl-3,6-diol (29). These observations suggest that reduced accumulation of this toxic sterol intermediate may contribute to the resistance phenotype of GET mutants by alleviating azole-induced sterol toxicity. However, the molecular link between GET-dependent protein trafficking and this sterol remodeling remains unresolved.

Sterols synthesized in the ER reach the plasma membrane via vesicular transport or membrane contact sites (MCSs) (70–72), the latter requiring oxysterol-binding proteins (OSBPs) as lipid transporters. Inhibition of OSBP activity results in sterol retention within the ER, leading to enhanced esterification and storage in lipid droplets (73). Interestingly, we found that Get-3 interacts with OSB-2 and OSB-6 (Table S3). Moreover, under KTC stress, GET deficiency led to reduced expression of *osb-4* compared with the wild-type (Table S6), a change that may be relevant to altered sterol distribution and membrane remodeling.

Our study identifies three possible critical GET-dependent cargoes, Emp-47, Cox-4, and Cox-15, which mediate azole resistance. Previous works in *Aspergillus fumigatus* and yeast have established a role of Emp-47 in glycoprotein trafficking (54, 74), and studies in yeast have characterized the Emp-47–Ssp120 complex as a receptor in the secretory pathway (74). Our additional analyses of the *emp-47* deletion mutant revealed extensive basal transcriptional remodeling involving translation, mitochondrial, and energy metabolism, and cell-surface-associated processes, suggesting that loss of Emp-47 alters cellular homeostasis before azole exposure. Notably, this phenotype was not accompanied by increased *erg11* or *cdr4* expression, reduced intracellular KTC accumulation, or detectable changes in sterol abundance, suggesting that the contribution of Emp-47 to azole susceptibility is unlikely to be explained by these canonical mechanisms alone.

We further connect the GET pathway with mitochondrial function through the identification of Cox-4 and Cox-15 as Get-3-interacting proteins (Fig. 3B and C). Moreover, deletion of multiple additional components associated with mitochondrial complex IV similarly reduced susceptibility to KTC (Fig. 3E), extending previous findings in *A. fumigatus* that dysfunction of respiratory complex IV components, including Cox7c and Cox10, promotes azole resistance (50, 59). Previous studies have linked complex IV dysfunction to altered ROS production, heme homeostasis, and calcium signaling, providing potential mechanisms through which defective Cox targeting may contribute to the azole resistance of GET mutants. The GET pathway may also promote mistargeting of mitochondrial outer membrane proteins (OMPs) to the ER (75), with ergosterol levels influencing TA protein sorting (76). Msp1/Doa10 extracts mislocalized OMPs, while GET mediates their ER retrieval (77). Together, these findings suggest a functional connection between GET-dependent protein targeting, mitochondrial complex IV function, and fungal adaptation to azole stress.

We discovered previously uncharacterized azole resistance-associated genes (e.g., *NCU00796*, *NCU07311*, and *NCU06911*; Fig. 4E) through transcriptome analysis and phenotype identification. These findings provide further mechanistic insight into the regulation of fungal azole susceptibility.

In summary, the functional and mechanistic model of GET pathway-mediated fungal azole resistance proposed in this study provides novel insights into the genetic basis of fungal drug resistance, and identifies candidate factors for further investigation of fungal azole susceptibility.

### Conclusion

This study identifies the GET pathway as a novel and conserved regulator of azole susceptibility in filamentous fungi. GET deficiency was not associated with reduced intracellular drug accumulation or azole-target overexpression; instead, it was associated with reduced accumulation of the toxic sterol intermediate 14α-methyl-3,6-diol under azole stress. Furthermore, GET deficiency perturbs the trafficking of multiple azole resistance-associated proteins, including the key tail-anchored protein transporter Emp-47 and mitochondrial Cox subunits, which are involved in vesicular transport and mitochondrial respiration. Accompanying this, GET disruption reprograms the expression of azole resistance-associated genes, independent of canonical stress responses. In this study, 19 previously uncharacterized genes modulating azole susceptibility are uncovered. Collectively, our work identifies the GET pathway as a non-classical regulator of azole susceptibility and reveals accompanying changes in protein trafficking, sterol metabolism, and resistance-associated gene expression, providing insights into non-classical antifungal resistance.

## Data Availability

The RNA-seq and mass spectrometry proteomics data supporting the results of this article are included in the supplemental material. The raw RNA-seq sequencing data have been deposited in the Gene Expression Omnibus (GEO) database under accession number GSE328637 and are publicly available. The mass spectrometry proteomics data have been deposited to the ProteomeXchange Consortium (https://proteomecentral.proteomexchange.org) via the iProX partner repository with the data set identifier PXD076447.

## SUPPLEMENTAL MATERIAL

Supplemental Figures mbio.02021-26-s0001.docx Fig. S1 to S5.

Table S1 mbio.02021-26-s0002.xlsx Strain characteristics and MICs.

Table S2 mbio.02021-26-s0003.xlsx Tail-anchored proteins in *Neurospora crassa*.

Table S3 mbio.02021-26-s0004.xlsx Get-3-interacting proteins identified by proximity-labeling proteomics.

Table S4 mbio.02021-26-s0005.xlsx RNA-seq analysis of the wild type and *emp-47^ko^* mutants with and without azole treatment.

Table S5 mbio.02021-26-s0006.xlsx A comparison of stress-responsive gene expression between the wild type and *emp-47^ko^* mutants.

Table S6 mbio.02021-26-s0007.xlsx RNA-seq analysis of the wild type and GET pathway mutants with and without azole treatment.

Table S7 mbio.02021-26-s0008.xlsx Comparison of stress-responsive gene expression between the wild type and GET pathway mutants.

Table S8 mbio.02021-26-s0009.xlsx Differentially expressed genes as key contributors to azole resistance in GET pathway mutants.

ASM does not own the copyrights to Supplemental Material that may be linked to, or accessed through, an article. The authors have granted ASM a non-exclusive, world-wide license to publish the Supplemental Material files. Please contact the corresponding author directly for reuse.
