# Supplementary material for: The GET pathway modulates azole susceptibility through sterol metabolic remodeling and protein trafficking in filamentous fungi
Source: mBio. 2026 Oct 7;17(11):e02021-26. doi: 10.1128/mbio.02021-26 (PMC13753843; doi:10.1128/mbio.02021-26)
Supplement: Supplementary file 1 [file mbio.02021-26-s0001.docx]

**Supplementary Figures**

**Figure S1 GET pathway contributes to sporulation and azole susceptibility.** (A) Sporulation efficiency of wild-type (WT) and GET pathway mutant strains. Representative slant cultures (left) and quantification of sporulation (right) for WT, *get-3*^KO^, *get-4*^KO^, and the corresponding complemented strains (*get-3*^oe-myc^ and *get-4*^COM^). Spores were harvested from slant cultures and counted. Data are presented as mean ± SD from three independent experiments. Statistical significance was determined by One-Way ANOVA followed by a Tukey's test. (B) Plate-based azole susceptibility assays of *A. fumigatus* strains *Af293* (FGSC#A1100) and the clinical isolate *Afget-4*^E46A^, which carries an E46A point mutation in *Afget-4*. Af293 was used as a reference strain. All experiments were performed with at least three independent biological replicates.

**Figure S2 Construction and functional validation of a TurboID‑Get-3 proximity‑labeling system.** Construction and validation of a TurboID-based proximity‑labeling system. (A) Plate assays of WT cultures grown with or without 1.5 µg/mL KTC in the presence of 0, 1, 4, or 8 µM biotin. Biotin did not alter WT susceptibility to KTC at the concentrations tested. A concentration of 4 µM biotin was selected for subsequent TurboID labeling (B) Schematic of the TurboID‑Get-3 fusion and principle of action in vivo. TurboID fused to the N‑terminus of Get-3 biotinylates proteins within a approximately 10‑nm radius in living cells, enabling their enrichment and identification by streptavidin‑based affinity capture. (C) A multidrug resistance phenotype was determined by drug susceptibility assay on plates. The TurboID‑Get‑3 strain exhibited azole sensitivity indistinguishable from that of the WT at the indicated azole concentrations. Plates were incubated at 28 °C for specified durations. Antifungal drugs used: KTC (ketoconazole), VRC (voriconazole), ITA (itraconazole).

**Figure S3** **Transcriptomic alterations associated with stress responses and ketoconazole susceptibility in the *emp-47*^ko^ strain**. (A–D) showing log_2_(FPKM + 1)-transformed expression levels of representative genes involved in (A) the reactive oxygen species (ROS) response, (B) the HSP90 pathway, (C) calcium signaling, and (D) detoxification processes. C and K indicate control and KTC-treated conditions, respectively. The underlying data are provided in Table S5. Statistical significance was assessed using a parametric Student’s t-test. ns, not significant; **P* < 0.05; ***P* < 0.01. (E) GO enrichment analysis of genes upregulated (purple) and downregulated (blue) in the *emp-47*^ko^ strain relative to WT under control conditions. Enriched biological process terms are shown. The corresponding GO enrichment data are provided in Table S4. (F) Heatmap of differentially expressed genes potentially associated with ketoconazole susceptibility in the *emp-47*^ko^ strain.

**Figure S4** **Effects of *emp-47* deletion on azole-response gene expression, ergosterol metabolism, and intracellular ketoconazole accumulation.** (A, C) Relative expression levels of *erg11* (A) and *cdr4* (C) in WT and *emp-47*^ko^ strains under control and KTC-treated conditions, based on RNA-seq data. (B) Relative ergosterol levels in WT and *emp-47*^ko^ strains with or without KTC treatment. (D) Relative intracellular KTC accumulation in WT and *emp-47*^ko^ strains following KTC treatment. (E) Relative levels of lanosterol, eburicol, and 14α-methyl-3,6-diol in WT and *emp-47*^ko^ strains with or without KTC treatment. Data are presented as mean ± SD (n = 3). Statistical significance in panels A–C and E was determined by two-way ANOVA followed by Tukey’s test; panel D was analyzed using Student’s t-test. **p* < 0.05; ns, not significant.

**Figure S5** **Transcriptional responses of ROS, HSP90, calcium signaling, and detoxification genes in GET pathway mutants under ketoconazole stress. (**A–D) showing log2(FPKM + 1)-transformed expression levels in WT, *get-3*^ko^, and *get-4*^ko^ strains for representative stress-response genes involved in in (A) the ROS response, (B) the HSP90 pathway, (C) calcium signaling, and (D) detoxification processes. C and K indicate control and KTC-treated conditions, respectively. The original data provided in Table S8. The significance of the difference was determined by a parametric Student’s t-test; **p* < 0.05, ns, not significant.
